# 5-Aminolaevulinic Acid-Mediated Photodynamic Therapy Combined with Tirapazamine Enhances Efficacy in Ovarian Cancer

**DOI:** 10.3390/biomedicines13030724

**Published:** 2025-03-16

**Authors:** Qian Wang, Yuping Suo, Xiaojuan Tian

**Affiliations:** 1 Fifth Clinical Medical College, Shanxi Medical University, Taiyuan 030012, China; wangqianx1026@163.com (Q.W.); 18435147092@163.com (X.T.); 2 Department of Gynaecology and Obstetrics, Shanxi Provincial People’s Hospital, Taiyuan 030012, China

**Keywords:** ovarian cancer, photodynamic, 5-aminolevulinic acid, hypoxia, tirapazamine

## Abstract

**Objectives**: Ovarian cancer is a common gynaecological malignancy. Photodynamic therapy (PDT) mediated by 5-aminolaevulinic acid (5-ALA-PDT) is widely used in clinical practice. However, hypoxia may impact the efficacy of this treatment. In the present study, we combined the bioreductively active drug tirapazamine (TPZ) with PDT to explore its potential in enhancing ovarian cancer cell death. **Methods**: A cell counting kit-8 assay was used to determine cytotoxicity under different intervention conditions. The distribution of protoporphyrin IX, a metabolite of 5-ALA, was observed using in vivo fluorescence imaging. The effect of the combined treatment was assessed by measuring changes in tumour size following the corresponding interventions and by haematoxylin and eosin staining of tumour tissues. Immunohistochemical staining was used to detect the expression levels of relevant proteins. **Results:** TPZ exhibited no cytotoxicity under normoxic conditions but was activated under hypoxic conditions, inducing cytotoxic effects that were enhanced when combined with PDT. Over time, protoporphyrin IX achieved systemic distribution, and high drug concentrations were maintained within the tumour. The combination therapy suppressed tumour growth, and pathological staining showed that necrotic tumour areas were significantly enlarged after treatment. The enhanced therapeutic effect may be attributable to the inhibition of the hypoxia-inducible factor-1α/vascular endothelial growth factor axis and PI3K/Akt/mTOR pathway. **Conclusions**: 5-ALA-PDT combined with TPZ can overcome both the hypoxic state of ovarian cancer tissues and the increased hypoxia induced by PDT, thereby inhibiting tumour growth.

## 1. Introduction

Ovarian cancer is the eighth most common cancer among women worldwide, accounting for 3.7% of cancer cases and 4.7% of cancer deaths annually, according to statistics from 2020 [1]. Globally, approximately 314,000 women are diagnosed with ovarian cancer each year, among whom 207,000 die [2]. Currently, surgery and chemotherapy are the primary clinical treatments for ovarian cancer. However, clinical outcomes and prognoses remain poor [3]. However, in recent years, novel therapeutic approaches have emerged [4].

Photodynamic therapy (PDT) is characterised by low toxicity, high selectivity, and repeatability, making it an effective treatment for many malignant diseases [5,6]. PDT combines photosensitisers or precursors with lights of specific wavelengths to generate reactive oxygen species, which induce cellular damage in the presence of oxygen [7]. Additional studies have confirmed that PDT’s specific mechanism of damage is related to apoptosis [8,9,10]. 5-aminolaevulinic acid (5-ALA) is the precursor of the photosensitiser protoporphyrin IX (PpIX). Although 5-ALA itself is not photosensitising, it is absorbed and metabolised into PpIX, which exhibits photosensitising properties [11]. The photodynamic reaction is highly efficient under laser irradiation at wavelengths corresponding to the photosensitisers, generating large quantities of reactive oxygen molecules [11]. Currently, 5-ALA-PDT is an effective treatment for several malignant tumours.

Hypoxia is a characteristic feature of tumours [12,13], arising from an imbalance between oxygen supply and consumption. Hypoxia is closely associated with drug resistance and tumour metastasis, compromising the effectiveness of many cancer therapies [14,15]. Moreover, oxygen, as an important factor in PDT, plays a crucial role [16]. Therefore, within the hypoxic environment of tumour tissue, the oxygen-consuming nature of PDT may affect its therapeutic efficacy. To address this issue, we identified a hypoxia-activated prodrug, tirapazamine. Tirapazamine (TPZ) is a novel bioreductive cytotoxic drug. Under aerobic conditions, the free radicals produced by these drugs via oxidoreductases are reoxidised and rendered inactive. By contrast, under hypoxic conditions, TPZ is metabolised by reductases into reactive free radicals, which induce DNA single- and double-strand breaks, ultimately leading to cell death [17,18]. This type of drug leverages the hypoxic tumour environment to exert potent antitumour effects. Therefore, we investigated the combination of TPZ with PDT to overcome hypoxia-related limitations and enhance PDT efficacy.

In this study, 5-ALA-PDT and TPZ were applied together in vivo to confirm that TPZ can mitigate tumour hypoxia, including hypoxia induced by PDT, and inhibit tumour growth. This study provides a new approach to addressing tumour hypoxia and improving the efficacy of PDT.

## 2. Materials and Methods

### 2.1. Cell Lines and Cell Culture

The human ovarian cancer cells (line OVCAR3) were purchased from FuHeng Biology (NO.202001027-04, Shanghai, China). A short tandem repeat (STR) analysis was performed to authenticate the cell line (EV value = 0.94). The cells were tested and confirmed to be free of Mycoplasma spp. OVCAR3 cells were maintained in a complete RPMI-1640 medium (Hyclone, Logan, UT, USA) supplemented with 20% foetal bovine serum (Cellmax, Beijing, China) and 1% streptomycin-penicillin (Beijing Solarbio Science & Technology Co., Ltd., Beijing, China) at 37 °C in a humidified atmosphere containing 5% CO_2_ and 95% air. To simulate hypoxic conditions, OVCAR3 cells were cultured in an environment containing 1% O_2_, 5% CO_2_, and 94% N_2_.

### 2.2. Chemicals and PDT Treatment

Next, 5-ALA (MedChemExpress, Shanghai, China) was prepared as an isotonic sodium chloride solution, and TPZ (MedChemExpress, Shanghai, China) was prepared as a solution in PEG300 (MedChemExpress, Shanghai, China). Both solutions were freshly prepared as needed. A light-emitting diode phototherapy device (Leirui Optoelectronic, Technology Co., Ltd., Changchun, China) with a peak emission wavelength of 633 ± 10 nm was used for PDT. During light treatment, the laser device was positioned approximately 2 cm from the cell layer or tumour to ensure that the light spot covered the target area.

### 2.3. Cell Counting Kit-8 Assay

Approximately 1.0 × 10^4^ cells were seeded into 96-well plates and cultured under either normoxic or hypoxic conditions. Cells under each condition were divided into four groups: control, TPZ, 5-ALA-PDT, and combined intervention. Cells designated for 5-ALA-PDT treatment were incubated with 5-ALA (1 mmol/L) for 4 h. Simultaneously, cells requiring TPZ treatment were incubated with TPZ (80 μmol/L). Cells requiring irradiation were exposed to light-emitting diode light at 633 ± 10 nm with an intensity of 20 mW/cm^2^ to achieve a total energy dose of 1.25 J/cm^2^. Subsequently, 10 μL of a WST-8 reagent (Boster Biological Technology, Co., Ltd., Wuhan, China) was added to each well. Absorbance was measured at 450 nm using a microplate reader.

### 2.4. Animals and Tumours

Female BALB/c athymic nude mice aged 4–5 weeks and weighing 17–22 g (n = 20) were used for this study. The mice were purchased from GemPharmatech Co., Ltd. (Changzhou, China) and housed in a specific pathogen-free (SPF) barrier facility at the Animal Centre of the Otolaryngology Head and Neck Surgery Laboratory at the First Hospital of Shanxi Medical University. All experiments were conducted in accordance with the ethical guidelines of the Fifth Hospital of Shanxi Medical University.

To establish subcutaneous tumours, 3 × 10^6^ OVCAR3 cells suspended in 200 μL of PBS were injected into the right flanks of the mice. After 6 days, subcutaneous tumour formation was observed, with the tumours measuring approximately 5–6 mm in diameter. The mice were randomly assigned to one of four groups: PDT treatment alone, TPZ treatment alone, combined PDT and TPZ treatment, or the control group. We selected the drug concentrations used in the experiments by reviewing the relevant literature [19,20,21,22]. TPZ (60 mg/kg) was administered via intraperitoneal injection, whereas 5-ALA (80 mg/kg) was administered intravenously via the tail vein. Control group mice received equal volumes of an isotonic sodium chloride solution. After drug administration, the mice were kept under dark conditions for 4 h, after which they were irradiated with red light (635 ± 5 nm, 40 J/cm^2^, 200 mW/cm^2^). Treatments were administered on alternate days, and tumour growth was measured every 2 days using callipers. Tumour volume was calculated using the following formula: 1/2 (length × width^2^). The formula for calculating the tumour volume in mice was presented in a previous study [23]. The accuracy of these formulas was evaluated by measuring the tumour volume in 50 mice. One of the formulas was ultimately confirmed to be the most accurate; V = C × L (the longest dimension) × W (the shorter dimension) × H (the height), where C = π/6 or 1/2. However, it was challenging to measure the height of such tumours, which grow under and are partially attached to the skin. Using the formula V = 1/2 × L × W^2^, we were able to determine the true tumour volume. The experiment was terminated when tumours reached 2000 mm^3^, at which point the animals were euthanised by inhaling CO_2_.

The experiments were conducted in accordance with the Guidelines for the Care and Use of Laboratory Animals (National Institutes of Health, Bethesda, MD, USA) and the project was approved by the Ethics Committee of Shanxi Provincial People’s Hospital (approval number 20230320).

### 2.5. In Vivo Fluorescence Imaging

The mice were anesthetised with isoflurane (with a concentration of 3% and a maintenance concentration of 1.5–2%) and then transferred to a PerkinElmer IVIS (Shanghai, China) imaging system to obtain fluorescence images at 1, 2, and 4 h after 5-ALA injection. All groups were imaged using an excitation wavelength of 430 nm and an emission wavelength of 640 nm. Fluorescence image quantification was performed using the IVIS system Living Image 4.4 software.

### 2.6. Haematoxylin and Eosin (HE) Staining

A portion of each tumour was fixed in 3.7% formaldehyde to embed paraffin. Serial sections (4 μm thick) were prepared from each specimen and mounted on glass slides. HE staining was performed on one section per specimen. The sections were first deparaffinized by treating them sequentially in xylene and graded alcohol (100%, 95%, 70%) to remove paraffin and hydrate the tissue. Next, the sections were stained with haematoxylin for 5–10 min, rinsed in tap water, and differentiated with 1% acid alcohol for a few seconds before being rinsed with water to return to blue. The tissue was then stained using eosin for 1–3 min and rinsed with distilled water to remove excess stain. This process was followed by dehydration through a gradient of alcohol, with transparency achieved using xylene. Finally, the slices were sealed with neutral gum and then observed under a microscope.

### 2.7. Immunohistochemical Analysis

Immunostaining of paraffin sections was performed after dewaxing and rehydrating 4 μm thick sections. The sections were dewaxed twice with dimethylbenzene and placed in absolute ethyl alcohol, graded ethanol, and distilled water. Antigen retrieval was performed using microwave treatment in 0.01 M sodium citrate buffer (Beijing Solarbio Science & Technology Co., Ltd., Beijing, China) at 95 °C for 15 min. For detection, the slides were incubated overnight at 4 °C with specific primary antibodies, including rabbit anti-human hypoxia-inducible factor (HIF)-1α antibody (1:100, Abclonal, Wuhan, China), vascular endothelial growth factor (VEGF) antibody (1:100, Proteintech, Wuhan, China), Bcl2 antibody (1:100, Abclonal, Wuhan, China), Bax antibody (1:100, Abcam, Cambridge, UK), Cleaved Caspase-3 (1:100, Abcam, Cambridge, UK), PI3K antibody (1:500, Abclonal, Wuhan, China), p-PI3K antibody (1:200, Abcam, Cambridge, UK), AKT antibody (1:200, Proteintech, Wuhan, China), p-AKT antibody (1:200, Proteintech, Wuhan, China), mTOR antibody (1:50, Cell Signalling Technology, Danvers, MA, USA), and p-mTOR antibody (1:200, Abcam, Cambridge, UK). The sections were then incubated with a horseradish-peroxidase-conjugated goat anti-rabbit secondary antibody (ZSGB-BIO, Beijing, China), following the manufacturer’s instructions. After washing again, the colour was developed using colourants such as DAB, and the development time was controlled under a microscope. Finally, the nuclei were re-stained with haematoxylin, dehydrated, made transparent, and sealed to complete the immunohistochemical staining. Next, the nuclei were observed under a microscope. Four fields of view per section were averaged to minimise errors. Cells stained significantly more intensely than the background staining with a dark brown colour and uniform and consistent intensity were classified as positive cells. The intensity and density of positively immunostained cells from different slides in each group were analysed using ImageJ software (version 1.54; National Institutes of Health, Bethesda, MD, USA).

### 2.8. Data Analysis

All statistical analyses were performed using GraphPad Prism 9.0.0. Immunohistochemical images were quantified using ImageJ software version 1.54. Overall data from multiple groups were compared using a one-way analysis of variance, followed by Dunnett’s post hoc test. The levels of significance were set as follows: * *p* < 0.05, ** *p* < 0.01, *** *p* < 0.001, **** *p* < 0.0001.

## 3. Results

### 3.1. TPZ Enhanced PDT Cytotoxicity Under Hypoxic Conditions

**Figure 1:**
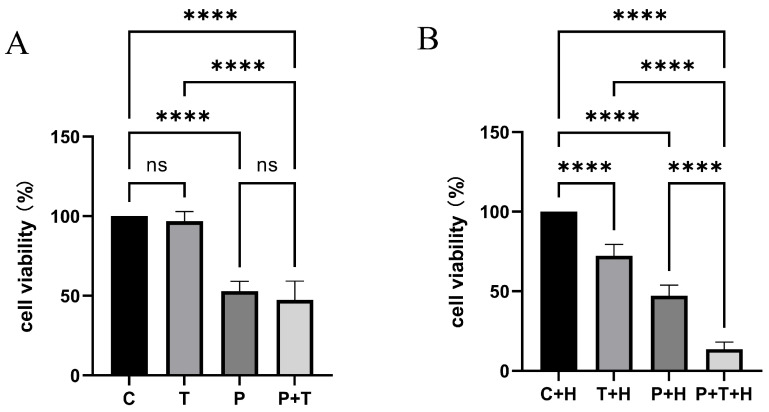
Cell counting kit-8 (CCK-8) assay results illustrating cytotoxicity under normoxic and hypoxic conditions. (**A**) Under normoxic conditions, PDT exhibited significant cytotoxicity. In contrast, TPZ alone was not cytotoxic, and combining TPZ with PDT did not enhance PDT cytotoxicity. C = control, T = TPZ alone, P = PDT alone, P + T = PDT combined with TPZ; ns, no significance, **** *p* < 0.0001. Data are presented as the mean ± standard error (n = 6). (**B**) Under hypoxic conditions, TPZ alone exhibited cytotoxicity. Combining TPZ with PDT further enhanced cytotoxicity. C + H = control under hypoxic conditions, T + H = TPZ alone under hypoxic conditions, P + H = PDT alone under hypoxic conditions, P + T + H = PDT combined with TPZ under hypoxic conditions; **** *p* < 0.0001. Data are presented as the mean ± standard error (n = 6). PDT, photodynamic therapy; TPZ, tirapazamine.

A cell counting kit-8 assay was used to determine cytotoxicity in OVCAR3 cells treated with a combination of 5-ALA-PDT and TPZ. TPZ alone exhibited no cytotoxicity under normoxic conditions. Furthermore, TPZ combined with PDT did not enhance PDT cytotoxicity, indicating that TPZ was not activated under normoxic conditions (Figure 1A). However, TPZ exhibited cytotoxic effects under hypoxic conditions, and cytotoxicity was further enhanced when combined with PDT (Figure 1B).

### 3.2. Combined Treatment Slowed Tumour Growth

**Figure 2:**
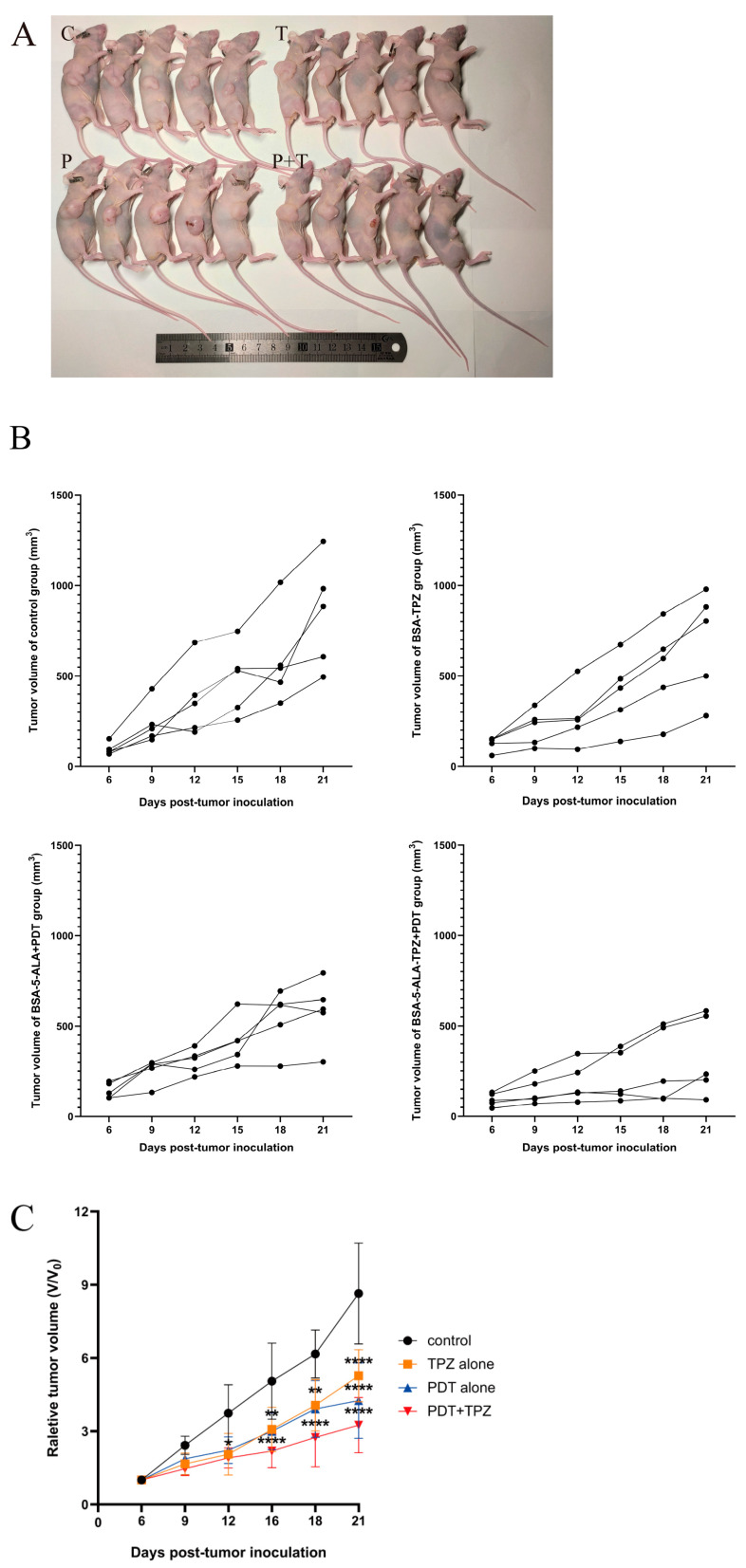
Changes in tumour volume in treated mice. (**A**) All nude mice were euthanised 21 d after tumour inoculation. C = control, P = PDT alone, T = TPZ alone, P + T = PDT combined with TPZ. (**B**) Tumour volume was measured and recorded before each treatment. Tumour growth curves were plotted for each mouse in each group. (**C**) Tumour growth curves after various treatments. Relative tumour volumes were normalised to their initial sizes. Combined treatment significantly inhibited tumour growth. * *p* < 0.05, ** *p* < 0.01, **** *p* < 0.0001. Data are presented as the mean ± standard error (n = 5). PDT, photodynamic therapy; TPZ, tirapazamine.

To investigate the antitumour effects of 5-ALA PDT combined with TPZ in vivo, we assessed its therapeutic efficacy in tumour-bearing mice. Tumour volume was recorded before each treatment to compare tumour growth rates during the treatment period. All three treatments inhibited tumour growth, with statistically significant differences compared to the control group. Notably, PDT combined with TPZ exhibited a statistically significant inhibitory effect on tumour growth by day 12 of the treatment. With prolonged treatment, tumour growth was significantly suppressed compared to that in the control group (Figure 2).

### 3.3. Combined Effect of 5-ALA-Mediated PDT and TPZ on Tumour Volume in Mice with Human Ovarian Cancer

We evaluated the combined effect by calculating changes in tumour volume in the mice. The nature of these combined effects was estimated using a previously published method [24,25,26]. Table 1 summarises the relative tumour volumes of the control and treatment groups at three different time points. The results indicated that 5-ALA-mediated PDT in combination with TPZ exerts a synergistic effect on tumour volume in mice.

### 3.4. PpIX Can Reach Systemic Distribution but Maintains High Concentrations Within the Tumour

**Figure 3:**
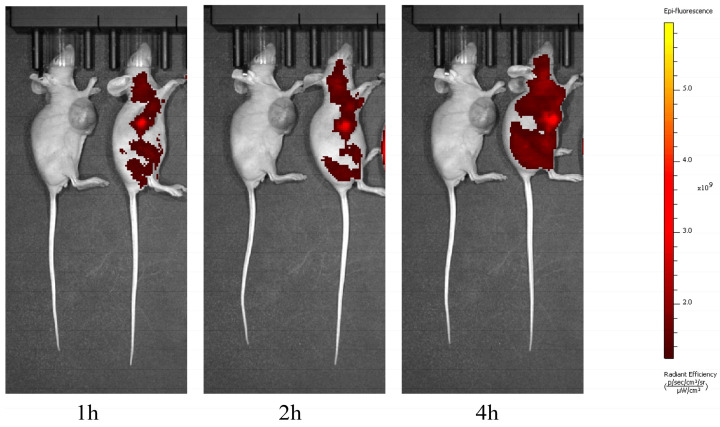
In vivo fluorescence imaging. Fluorescence distribution of PpIX was observed at 1, 2, and 4 h after 5-ALA injection. The left mice served as the blank control group, and the right mice received 5-ALA via tail vein injection. Results showed that PpIX can achieve systemic distribution over time but remains at high concentrations within the tumour for a certain period. PpIX, protoporphyrin IX; 5-ALA, 5-aminolaevulinic acid.

The distribution of PpIX was observed using in vivo fluorescence imaging at 1, 2, and 4 h after a tail vein injection of 5-ALA into the mice. Over time, PpIX gradually achieved systemic distribution. At 4 h, we observed that PpIX had reached systemic distribution but maintained a high concentration in the tumour (Figure 3). PpIX, a metabolite of 5-ALA, is an intermediate in the heme synthesis pathway. The toxicity of this metabolite is low, and high concentrations of PpIX can exert cytotoxic effects only when photosensitised via laser irradiation at appropriate wavelengths. Over time, PpIX achieved systemic distribution; therefore, we chose to irradiate the tumour locally in subsequent experiments to avoid damage to normal tissues.

### 3.5. HE Staining Revealed That the Combination Treatment Significantly Increased the Extent of Necrosis

**Figure 4:**
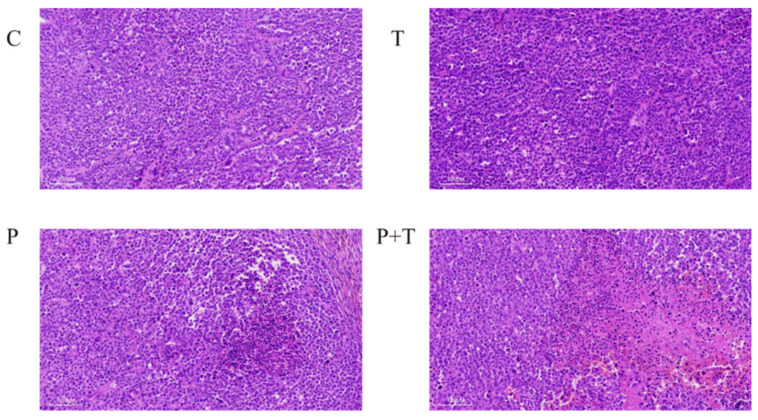
HE staining. The combination treatment significantly increased the necrotic area compared with that in monotherapy, as shown by HE staining. C = control, P = PDT alone, T = TPZ alone, P + T = PDT combined with TPZ (original magnification, ×100; scale bar, 100 μm). PDT, photodynamic therapy; TPZ, tirapazamine; HE, haematoxylin and eosin.

In this study, we examined the effects of different interventions using HE staining. After treatment with TPZ alone, scattered necrotic cells were observed in the tumour tissue. After treatment with PDT alone, obvious cell necrosis was observed. However, the necrotic area was limited. After combined PDT and TPZ treatment, a large area of diffuse necrosis was visible. Moreover, we found that haemorrhaging was more frequent after combination therapy than after monotherapy. However, further experiments are required to determine whether haemorrhaging is directly related to combination therapy (Figure 4).

### 3.6. Changes in the Expression Levels of Apoptosis-Related Proteins Indicate That Combination Therapy Enhanced Apoptosis

**Figure 5:**
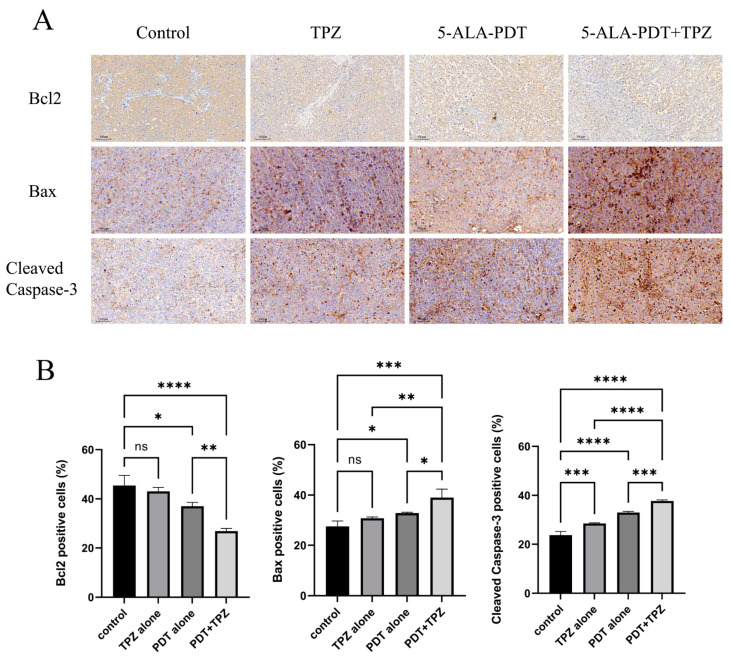
Immunohistochemical staining of apoptosis-related proteins in tumours. (**A**) Immunohistochemical staining showed changes in the number of apoptosis-associated protein-positive cells after combination therapy (original magnification, ×100; scale bar, 100 μm). (**B**) The number of Bcl-2-positive cells decreased sequentially from the control, TPZ, and PDT groups to the combination treatment group, whereas the number of Bax- and cleaved caspase-3-positive cells increased sequentially. ns, no significance, * *p* < 0.05, ** *p* < 0.01, *** *p* < 0.001, **** *p* < 0.0001. Data are presented as the mean ± standard error (n = 5). PDT, photodynamic therapy; TPZ, tirapazamine.

We confirmed protein expression levels in the tumours using immunohistochemical staining. The expression level of Bcl2 decreased sequentially in the control, TPZ alone, PDT alone, and combination treatment groups. By contrast, the expression levels of Bax and Cleaved Caspase-3 increased sequentially in the control, TPZ alone, PDT alone, and combination treatment groups. These results suggest that combination treatment enhanced apoptosis to some extent (Figure 5).

### 3.7. Expression Levels of HIF-1α/VEGF Axis-Related Proteins and Phosphorylated Proteins in the PI3K/Akt/mTOR Pathway Were Reduced in Tumours After Combination Therapy

**Figure 6:**
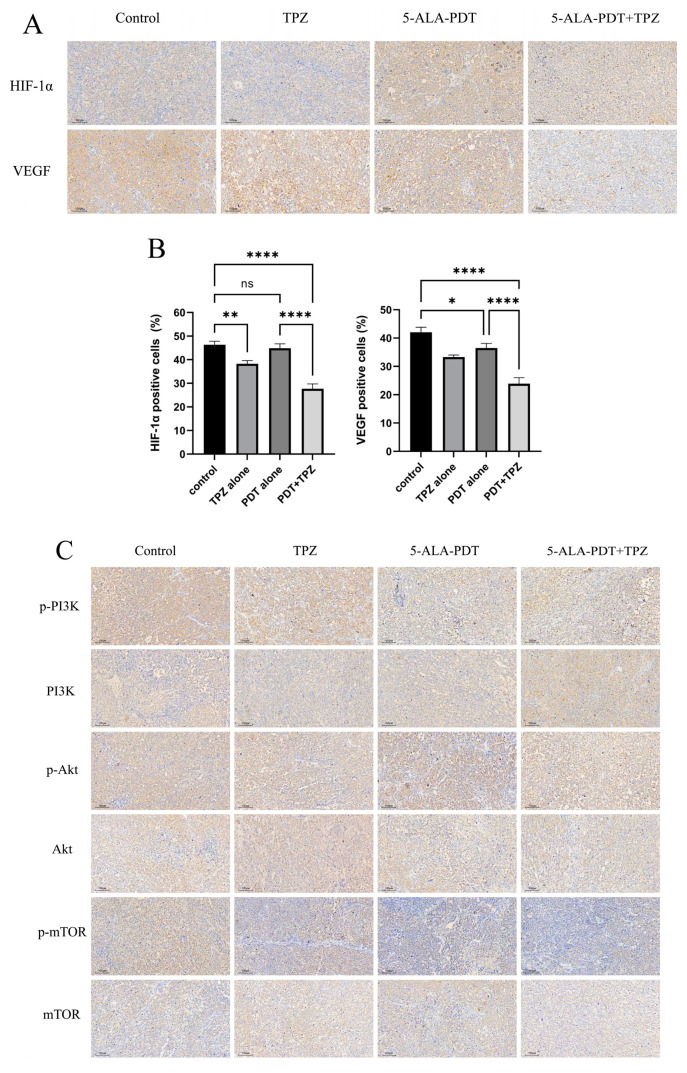

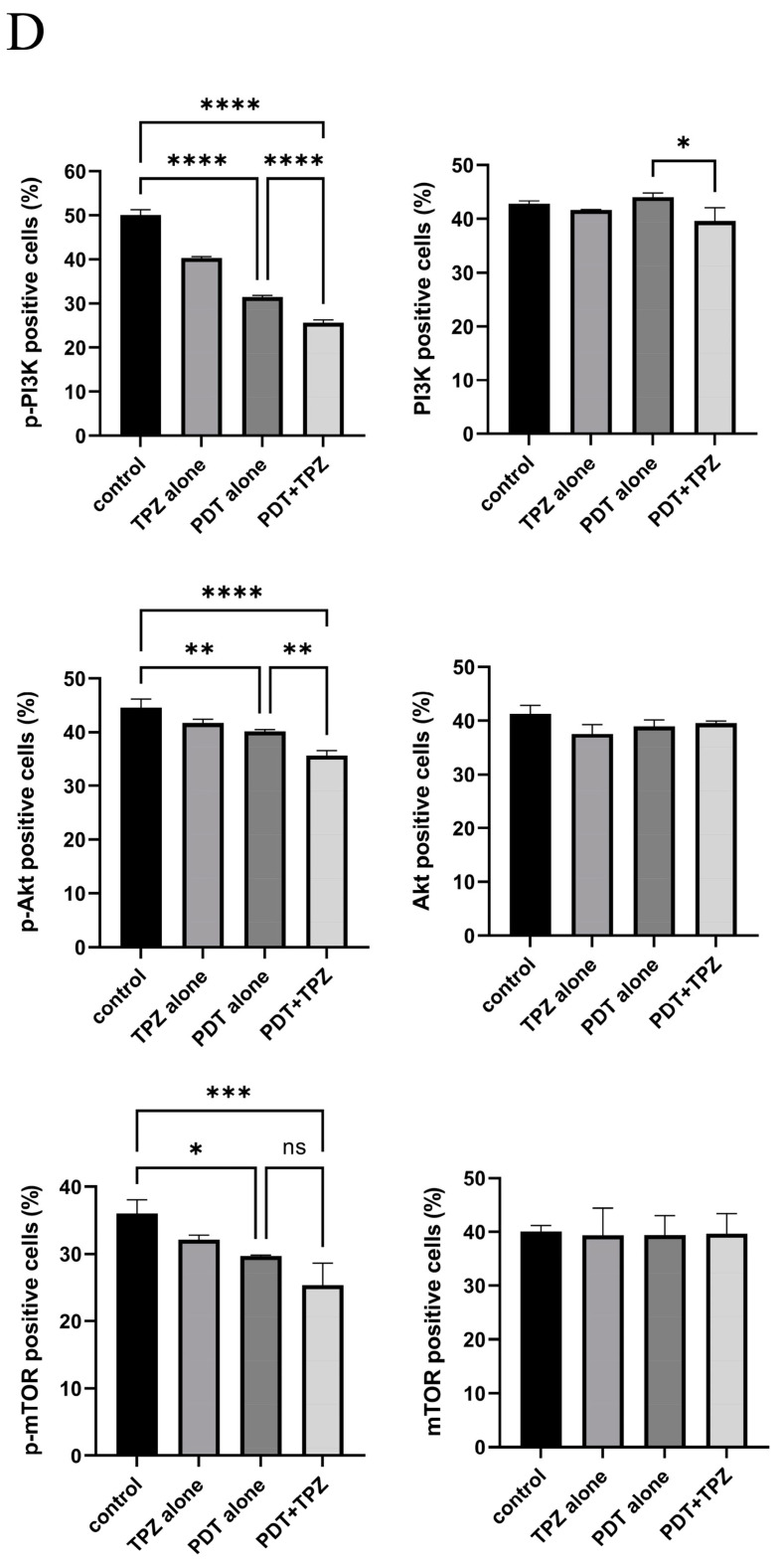
Immunohistochemical staining of relevant proteins in tumours. (**A**,**C**) Immunohistochemical staining showed that combination treatment reduced the number of protein-positive cells in tumours (original magnification, ×100; scale bar, 100 μm). (**B**) Combination therapy resulted in a decrease in HIF-1α and VEGF protein expression. ns, no significance, * *p* < 0.05, ** *p* < 0.01, **** *p* < 0.0001. Data are presented as the mean ± standard error (n = 5). (**D**) Combination therapy reduced the expression of phosphorylated proteins in the PI3K/Akt/mTOR pathway, suggesting that pathway activation was inhibited. ns, no significance, * *p* < 0.05, ** *p* < 0.01, *** *p* < 0.001, **** *p* < 0.0001. Data are presented as the mean ± standard error (n = 5). VEGF, vascular endothelial growth factor; HIF, hypoxia-inducible factor.

Similarly, the expression levels of HIF-1α and VEGF exhibited similar trends. PDT alone had minimal effects on hypoxia-associated protein expression compared with the control group. TPZ alone reduced the expression of these proteins. After combination therapy, the expression of hypoxia-associated proteins decreased further. The trends in the expression of PI3K/Akt/mTOR pathway-related proteins were also similar. Total protein expression did not significantly differ among groups. However, the expression of corresponding phosphorylated proteins decreased sequentially in the control, TPZ, PDT, and combination treatment groups (Figure 6).

## 4. Discussion

Light, photosensitisers, and oxygen are the three most important factors in PDT [27] and generate highly active singlet oxygen by transferring electrons to oxygen, thus leading to cell death [28]. However, because of several physiological changes in the tumour microenvironment [29], such as irregularities in the vascular system, uncontrolled proliferation of tumour cells, and deterioration of the microenvironment [30], hypoxia is a feature of most tumours. PDT is an oxygen-consuming process that can cause acute hypoxia within tumour tissues and promote vascular collapse while decreasing cellular oxygenation [31,32]. When PDT is applied to tumour tissues, its cytotoxic effect is affected by a shortage of oxygen. Therefore, we combined a hypoxia-activatable clinically used bioreductive prodrug, TPZ, with 5-ALA-PDT to utilise its specific cytotoxicity capabilities under low-oxygen environments. We sought to exploit the hypoxic characteristics of the tumour itself and the hypoxic environment caused by PDT to improve the therapy’s anticancer effects. In our study, we showed that using a combination of 5-ALA-PDT and TPZ enhanced anticancer activity.

In this study, we injected 5-ALA through the tail vein of the mice and found that 5-ALA showed systemic distribution but consistently maintained high concentrations in tumour tissues over time. Notably, 5-ALA selectively aggregates in tumour tissues. In this study, 5-ALA-PDT alone was applied to ovarian cancer in mice, and the results showed that tumour growth began to slow after 16 days of treatment compared to the results in the control group, and HE staining presented flaky necrotic areas in the tumour. These experimental results confirm that 5-ALA-PDT has a cytotoxic effect on ovarian cancer tissues, even under hypoxic conditions. Current research on the mechanism of PDT mainly focuses on the toxic effects of inducing apoptosis [33,34,35,36], which were also confirmed by our previous related studies [10,37]. In our experiments, we observed a decrease in the expression of apoptosis-regulatory protein Bcl2 and an increase in the expression of Bax and Cleaved Caspase-3 via immunohistochemical staining, which also confirmed the related role of apoptosis.

We used TPZ, a hypoxia-activated prodrug, which is activated and cytotoxic only in hypoxic environments [38]. Under hypoxia, TPZ is metabolised by intracellular reductase to form a highly reactive free radical capable of inducing single- and double-strand breaks in DNA. Under aerobic conditions, free radicals are de-oxidised to parent compounds. This cycling provides hypoxia selectivity [17,39,40]. Studies have also shown that TPZ can inhibit anti-apoptosis and radioresistance caused by the presence of hypoxic cells in tumour tissues, so it can significantly increase the anti-tumour effects of tumour radiotherapy [41,42]. In in vitro cellular experiments, TPZ was not activated under normoxic conditions. By contrast, significant cytotoxicity was observed under hypoxic conditions. In in vivo experiments, TPZ intervention alone resulted in scattered stellate necrosis within the tumour, which inhibited tumour growth on day 16 of treatment. Immunohistochemical analysis confirmed the presence of elevated Cleaved Caspase-3 protein expression after TPZ treatment, demonstrating that TPZ alone increased apoptosis. Based on the features of TPZ, we believe that in the hypoxic environment of tumour tissues, the application of PDT may increase hypoxia and activate TPZ [43], thus achieving synergistic effects and increasing cytotoxic effects. In in vivo experiments, tumour growth slowed from day 12 post-inoculation after combination treatment. Large patchy necrotic areas were observed in the tumour. The immunohistochemical results also illustrated that the combination treatment further increased apoptosis.

In addition, we focused on proteins and pathways associated with tumour proliferation and hypoxia. The PI3K/Akt/mTOR signalling pathway is an important pathway that regulates the cell cycle and is directly related to tumour growth, proliferation, and apoptosis [44]. Aberrant activation of this signalling pathway is common in cancer and plays an important regulatory role in cell survival, proliferation, and angiogenesis [45]. HIF-1α is regulated by the PI3K/Akt/mTOR signalling pathway [46,47]. PI3K inhibitors and PI3K/mTOR inhibitors can inhibit p-Akt activation and the expression of HIF-1α and VEGF [48,49]. Akt is an important proto-oncogene, a key downstream molecule in the growth factor signalling pathway, and mediates a variety of pro-proliferative and inhibitory apoptotic biological effects upon activation by PI3K [50]. Akt inhibitors inhibit the expression of HIF-1α at the protein level [44]. mTOR is a sensor of hypoxia and target of Akt in cell cycle regulation, glycogen metabolism, and protein synthesis, as well as an upstream mediator of HIF-1α activation [51,52]. In this study, we found that the levels of phosphorylated proteins in the PI3K/Akt/mTOR pathway decreased after a single intervention. An obvious reduction in phosphorylated protein expression was observed after the combination treatment, suggesting that a combination of 5-ALA-PDT and TPZ may inhibit activation of the PI3K/Akt/mTOR pathway, impeding its protective effects and leading to cell death.

Other hypoxia-associated markers include HIF and VEGF. HIF can accumulate intracellularly to regulate the tumour microenvironment and adapt to hypoxic conditions. HIF enhances tumour–stromal cell interactions under hypoxic conditions through its dependent signalling pathway, thereby promoting tumour progression [53]. The expression of HIF is regulated by many factors and represents a complex process. The correlation between the PI3K/Akt signalling pathway and HIF-1α under hypoxic conditions varies across different diseases and cell types. The stability of HIF-1α is related to the PI3K/Akt signalling pathway, and PI3K inhibitors can reduce the expression of HIF-1α [54,55,56,57]. Blockade of the PI3K/Akt/mTOR pathway inhibited its ability to induce HIF-1α expression but had no effect on hypoxia-induced HIF-1α expression [58]. Hypoxia not only activates the PI3K/Akt signalling pathway in cells but also promotes HIF-1α expression [59]. Moreover, hypoxia-induced HIF-1α activation seems to precede PI3K/Akt activation [60]. In our study, we found that although PDT treatment alone did not result in a reduction in HIF-1α expression within the tumour, the combination treatment of PDT with TPZ significantly reduced HIF-1α expression within the tumour. Meanwhile, enhanced HIF-1α expression under hypoxic conditions stimulated tumour cells to secrete VEGF [61]. Akt together with HIF-1α induced vascular endothelial growth factor expression and angiogenesis [62]. Angiogenesis is necessary for tumour growth and leads to tumour recurrence and metastasis by affecting the balance between pro- and anti-angiogenic factors [63]. Among the pro-angiogenic factors, VEGF is the most important. Angiogenesis plays a key role in disease progression and resistance. In vivo experiments in mice confirmed that the combination treatment significantly reduced the number of VEGF-positive cells in the tumour and the expression of VEGF. This result suggests that combination therapy may inhibit tumour growth and metastasis by suppressing tumour tissue angiogenesis and thus tumour growth and metastasis.

However, there are some limitations and shortcomings in our study. (1) Although we sought to ensure that the same number of cells was injected into each mouse during tumour implantation, the final tumour volume varied greatly due to individual differences and other factors. At the same time, for ease of measurement, we chose to measure only the long and short diameters. However, the tumours were actually irregular, and the standard formulas used to calculate the tumour volume were all based on the assumption that the tumour shape is approximately elliptical. (2) Although tumour hypoxia has been widely studied as one of the characteristics of tumours, it is still necessary to confirm the hypoxic environment of tumours experimentally through appropriate detection methods, such as pimonidazole staining. (3) In this study, we focused on the effects of combination therapy, as there are few studies on the effects of TPZ alone. (4) Although our experiments indicate that combination therapy has a stronger inhibitory effect than monotherapy on tumours, the mechanism of interaction between these two therapies requires further study. (5) Further long-term trials are needed to investigate the long-term effects of this combination therapy, its potential toxicities, and its impact on overall survival.

In summary, PDT’s exacerbation of the tumour hypoxic environment may provide conditions for the activation of TPZ, with the combination of 5-ALA-PDT and TPZ able to enhance the cytotoxic effects. This mechanism may be related to the inhibition of the HIF-1α/VEGF axis and PI3K/Akt/mTOR pathway. Other possible mechanisms of cytotoxicity should be investigated in future studies.

## Figures and Tables

**Table 1:** Synergistic effect of ALA PDT and TPZ.

Fractional Tumour Volume (FTV) Relative to Untreated Controls ^a^					
			Combination Treatment		
Day ^b^	5-ALA	TPZ	Expected ^c^	Observed	Ratio of Expected FTV/Observed FTV ^d^
15	1.00 ± 0.55	1.05 ± 0.67	1.05	0.44 ± 0.17	2.42
18	0.97 ± 0.26	1.14 ± 0.71	1.10	0.47 ± 0.34	2.33
21	0.71 ± 0.15	0.93 ± 0.50	0.66	0.37 ± 0.16	1.80

^a^ FTV = (mean tumour volume in the experimental group)/(mean tumour volume in the control group). ^b^ Day after tumour cell transplantation. ^c^ (Mean FTV of 5-ALA PDT) × (Mean FTV of TPZ). ^d^ A ratio of >1 indicates a synergistic effect, whereas a ratio of <1 indicates a less-than-additive or antagonistic effect. PDT, photodynamic therapy; TPZ, tirapazamine; 5-ALA, 5-aminolaevulinic acid.

## Data Availability

The data generated in the present study may be requested from the corresponding author.
